# Mothers know best: spatial and nutritional limitations shape offspring performance in the predator mite *Amblyseius herbicolus*

**DOI:** 10.1093/cz/zoag008

**Published:** 2026-02-13

**Authors:** Yuzhi Gong, Qiang Xuan, Keshi Zhang, Haiyu Chi, Lanjing Li, Junlin Cao, Zhi-Qiang Zhang

**Affiliations:** Manaaki Whenua – Landcare Research Group, Bioeconomy Science Institute, 231 Morrin Road, Auckland 1072, New Zealand; State Key Laboratory of Palaeobiology and Stratigraphy, Nanjing Institute of Geology and Palaeontology, Chinese Academy of Sciences, 39 East Beijing Road, Nanjing 210008, China; University of Chinese Academy of Sciences, 1 Yanqihu East Road, Beijing 100049, China; Manaaki Whenua – Landcare Research Group, Bioeconomy Science Institute, 231 Morrin Road, Auckland 1072, New Zealand; School of Biological Sciences, University of Auckland, 3A Symonds Street, Auckland 1010, New Zealand; Manaaki Whenua – Landcare Research Group, Bioeconomy Science Institute, 231 Morrin Road, Auckland 1072, New Zealand; School of Biological Sciences, University of Auckland, 3A Symonds Street, Auckland 1010, New Zealand; Manaaki Whenua – Landcare Research Group, Bioeconomy Science Institute, 231 Morrin Road, Auckland 1072, New Zealand; School of Biological Sciences, University of Auckland, 3A Symonds Street, Auckland 1010, New Zealand; Manaaki Whenua – Landcare Research Group, Bioeconomy Science Institute, 231 Morrin Road, Auckland 1072, New Zealand; School of Biological Sciences, University of Auckland, 3A Symonds Street, Auckland 1010, New Zealand; Manaaki Whenua – Landcare Research Group, Bioeconomy Science Institute, 231 Morrin Road, Auckland 1072, New Zealand; School of Biological Sciences, University of Auckland, 3A Symonds Street, Auckland 1010, New Zealand

**Keywords:** captivity, immature development, maternal influence, parental effect, transgenerational phenotypic plasticity

## Abstract

Rearing conditions play a crucial role in shaping the life-history traits of arthropods, yet the combined effects of rearing environment and prey availability within and across generations remain poorly understood. This study investigated how rearing cell size (large vs. small) and prey availability (30 vs. 60 *Carpoglyphus lactis* eggs per day) influence the performance of the predatory mite *Amblyseius herbicolus* and its offspring under controlled laboratory conditions. Within the maternal generation, individuals confined to small cells consumed significantly more prey but showed no improvement in development, survival, or adult size, indicating stress-related increases in energy expenditure. High prey availability enhanced survival rate, and its interaction with cell size revealed that high prey availability mitigated the negative effects of stress. Under high prey availability, mothers in large cells produced remarkably smaller eggs, suggesting altered reproductive investment under stress. Across generations, offspring of mothers reared under low prey availability and small cells showed higher survival and larger adult size, reflecting compensatory maternal effects. These findings partially support the hypothesis that restricted rearing environments and low prey availability negatively affect within-generation performance but promote adaptive transgenerational responses, providing new insight into maternal effects and rearing optimization in phytoseiid predators.

## Graphical Abstract

**Graphical Abstract:**
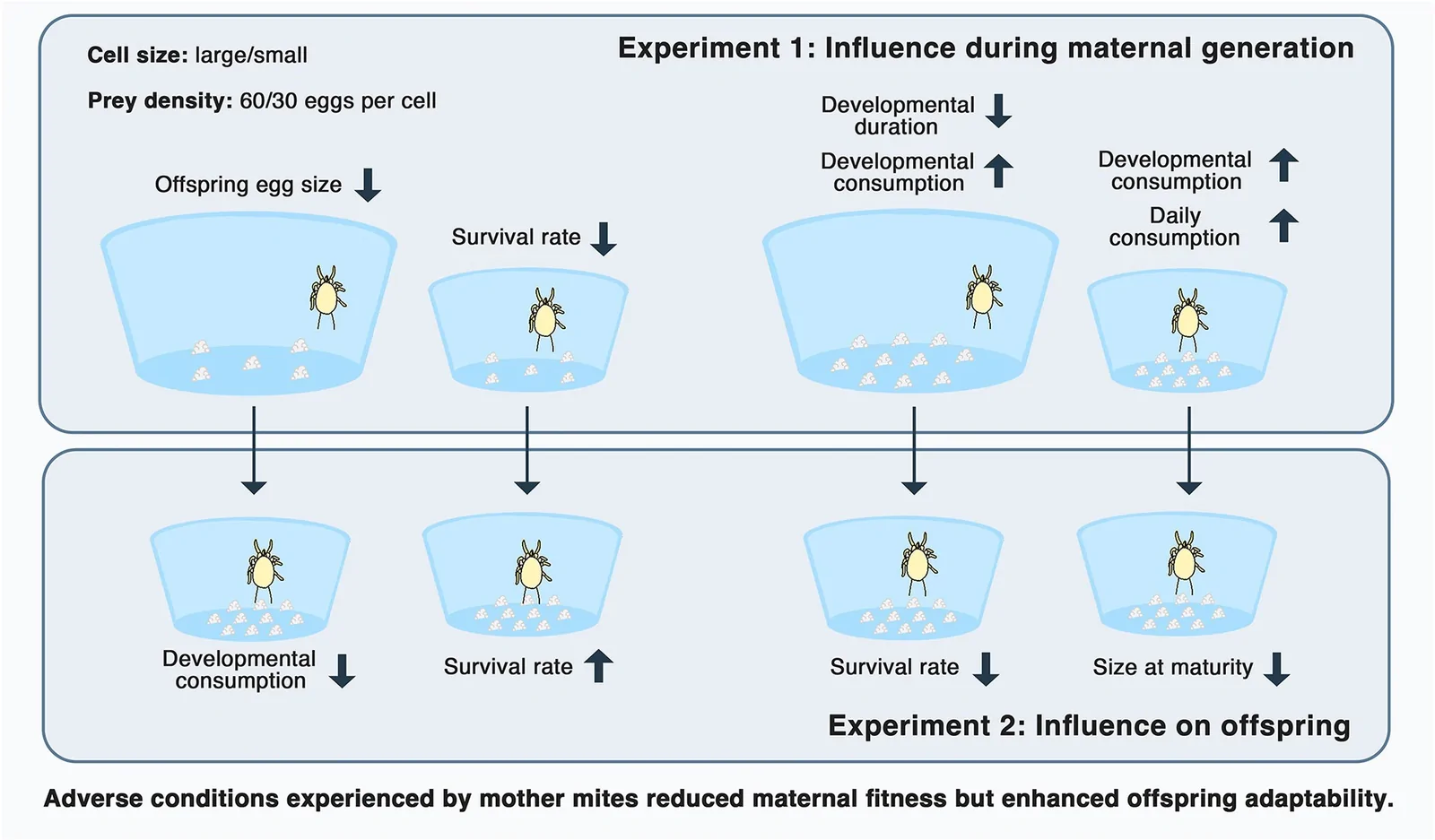

Captivity refers to the maintenance of animals in controlled environments such as laboratories, zoos, and other artificial rearing facilities, where their freedom is restricted and environmental conditions, such as space, diet, and social interactions, are manipulated (Mason 2010; Perdue et al. 2020). In captivity, spatial constraints, lack of choice, altered social structure, and modified complex level can induce chronic stresses that result in physiological, behavioral, and morphological changes in diverse animal species (Mason 2010). Moreover, stressful conditions can adversely affect multiple life-history traits, including reproduction and longevity (Morgan and Tromborg 2007; Perdue et al. 2020; Crates et al. 2023). For example, small cage size and high rearing density can negatively affect reproduction, growth, and offspring survival in captive animals, such as mice (An et al. 2021), farm chickens (Widowski et al. 2017), and birds (Bussolini et al. 2023).

Most studies have focused on vertebrate species, but stressful conditions related to limited space and high density also affect invertebrates (Pollo and Vitale 2019; Lewbart and Zachariah 2023). For example, the size of the captive enclosure can have crucial effects on the survival and reproduction of invertebrate species (Kavousi et al. 2009; Bosua et al. 2018). Many invertebrate species play important roles in human life and are widely used for agricultural, economic, and scientific purposes (Pollo and Vitale 2019), so it is important to understand these effects.

Maternal effects are influences of the maternal phenotype on offspring traits beyond direct genetic inheritance (Räsänen and Kruuk 2007). Both abiotic and biotic experiences encountered by the mother can be conveyed to the next generation through epigenetic mechanisms, including DNA methylation, post-translational histone modifications, and non-coding RNA expression (Bonduriansky and Day 2009; Bonduriansky 2012; Bell and Hellmann 2019; Anastasiadi et al. 2021). Maternal effects enable mothers to transmit information about past or current environmental conditions, thereby shaping offspring phenotypes in ways that may enhance fitness under anticipated environmental conditions (Kuijper and Johnstone 2018). In addition to such adaptive influences, the physiological state of the mother may affect offspring development through passive condition transfer, which may not necessarily reflect future environmental conditions (Bonduriansky et al. 2018). These maternal influences can have lasting impacts on the developmental trajectory of offspring, affecting traits related to growth, survival, and reproduction (Groothuis et al. 2020).

The family Phytoseiidae (Acari: Mesostigmata) includes predominantly free-living, plant-dwelling predatory mites, many of which play an important role in the biological control of agricultural pests (Zhang 2003a; McMurtry et al. 2015). Several species within this family, such as *Amblyseius swirskii*, *Neoseiulus cucumeris*, and *Phytoseiulus persimilis*, have been commercially mass-reared and widely implemented in biocontrol, targeting a variety of insect and mite agricultural pests (Amano and Chant 1977; Zhang 2003b; McMurtry et al. 2013). The successful establishment and long-term persistence of phytoseiid populations are crucial for sustainable pest suppression (Amano and Chant 1978; Xie et al. 2018; Xu et al. 2023). Considerable research has been dedicated to optimizing rearing protocols to improve the efficiency and reliability of these natural enemies in agricultural systems, but the effects of rearing cell size in invertebrate systems, including these predatory species, are rarely examined (Carere and Mather 2019). Moreover, many phytoseiid species have been extensively used in the study of ecology and evolution, including life history traits and maternal or parental effects, which often involves laboratory experiments utilizing enclosed arenas (Han et al. 2022, 2024; Xu et al. 2023, 2025; Zhang and Zhang 2025). Therefore, the influence of enclosure size on invertebrates, including phytoseiids, needs to be examined.

*Amblyseius herbicolus* (Chant, 1959) is a thelytokous (females reproduce without mating) phytoseiid predator with a wide geographic distribution and is regarded as a promising candidate for the biocontrol of various pests (Reis et al. 2007; Cavalcante et al. 2015; Kalile et al. 2021; Lam et al. 2021; Xin and Zhang 2021). In this study, we investigated the effect of rearing cell size (large vs. small) and prey availability (high vs. low) on the performance of *A. herbicolus* mothers and their offspring. Given that restricted environments may induce stress in mothers and alter their offspring provisioning, we proposed 2 hypotheses:

1. Mothers reared in smaller rearing cells and under lower prey availability would exhibit negative effects on their own life-history traits compared with mothers reared in larger rearing cells and under higher prey availability.
2. Offspring of mothers reared in smaller rearing cells and under lower prey availability would also show reduced performance in life-history traits compared with the offspring of mothers from larger rearing cells and higher prey availability.

Testing these hypotheses provides new insights into how spatial and nutritional conditions influence both within- and cross-generational performance in *A. herbicolus*. The findings have implications for optimizing mass-rearing protocols and improving the reliability of experimental systems involving phytoseiid predators and other invertebrate models.

## Materials and methods

### Mite cultures

The *A. herbicolus* individuals used in this study were established from adult females (>30) collected from avocado (*Persea americana* Mill.) leaves sourced from an orchard located in Te Puna, Tauranga, New Zealand. Species identification was confirmed based on the morphological description provided by Ma et al. (2024). The laboratory colony was maintained under controlled conditions and provisioned ad libitum with mixed-stage dried fruit mites, *Carpoglyphus lactis* (L.) (Acari: Carpoglyphidae), obtained from Bioforce Limited (Karaka, New Zealand). At the time of the experiment, the mite colonies had been maintained for more than 1 year.

Rearing and culturing procedures followed previously described methods, with slight modifications to unit dimensions (Zhang and Zhang 2021; Wang et al. 2024). All cultures and experimental units were housed within acrylic cabinets (100 × 55 × 100 cm; length × width × height) maintained at 25 ± 1 °C, 80 ± 5% relative humidity, and a 16:8 h light: dark photoperiod. Cultures were maintained in plastic containers (225 × 225 × 35 mm) filled with filtered water. Within each container, a Petri dish (55 mm diameter) was positioned on a black plastic sheet and supported by a water-soaked sponge (100 × 100 × 30 mm) to prevent overflow. The water served as both a hydration source and a barrier to prevent mite escape.

*Carpoglyphus lactis* cultures were maintained directly in the Petri dishes using a locally prepared substrate composed of approximately 96% wheat bran (Edmonds, New Zealand), 3% dry yeast (Edmonds, New Zealand), and 1% icing sugar (Chelsea, New Zealand). The substrate and mites were replenished regularly, and the water was refreshed weekly. Frozen *C. lactis* eggs were prepared according to Liu et al. (2024), stored at −18 °C for at least 1 week, and thawed at room temperature (c. 25 °C) for 30 min before use in experiments.

### Rearing cells

Two modified Munger-type cells of different sizes were used for experimental rearing (Zhang and Zhang 2021). The large cell comprised 2 plexiglass slides (38 × 38 × 3 mm), with the upper slide (Plexiglass I) featuring a centrally drilled hole (15 mm diameter; volume: 530.14 mm^3^) serving as the rearing chamber (Figure 1A). The small cell consisted of 2 plexiglass slides (38 × 25 × 2 mm), with the upper slide (Plexiglass II) containing a centrally located, truncated, cone-shaped hole (top diameter: 10 mm; bottom diameter: 7 mm; volume: 114.67 mm^3^) (Figure 1A).

**Figure 1:**
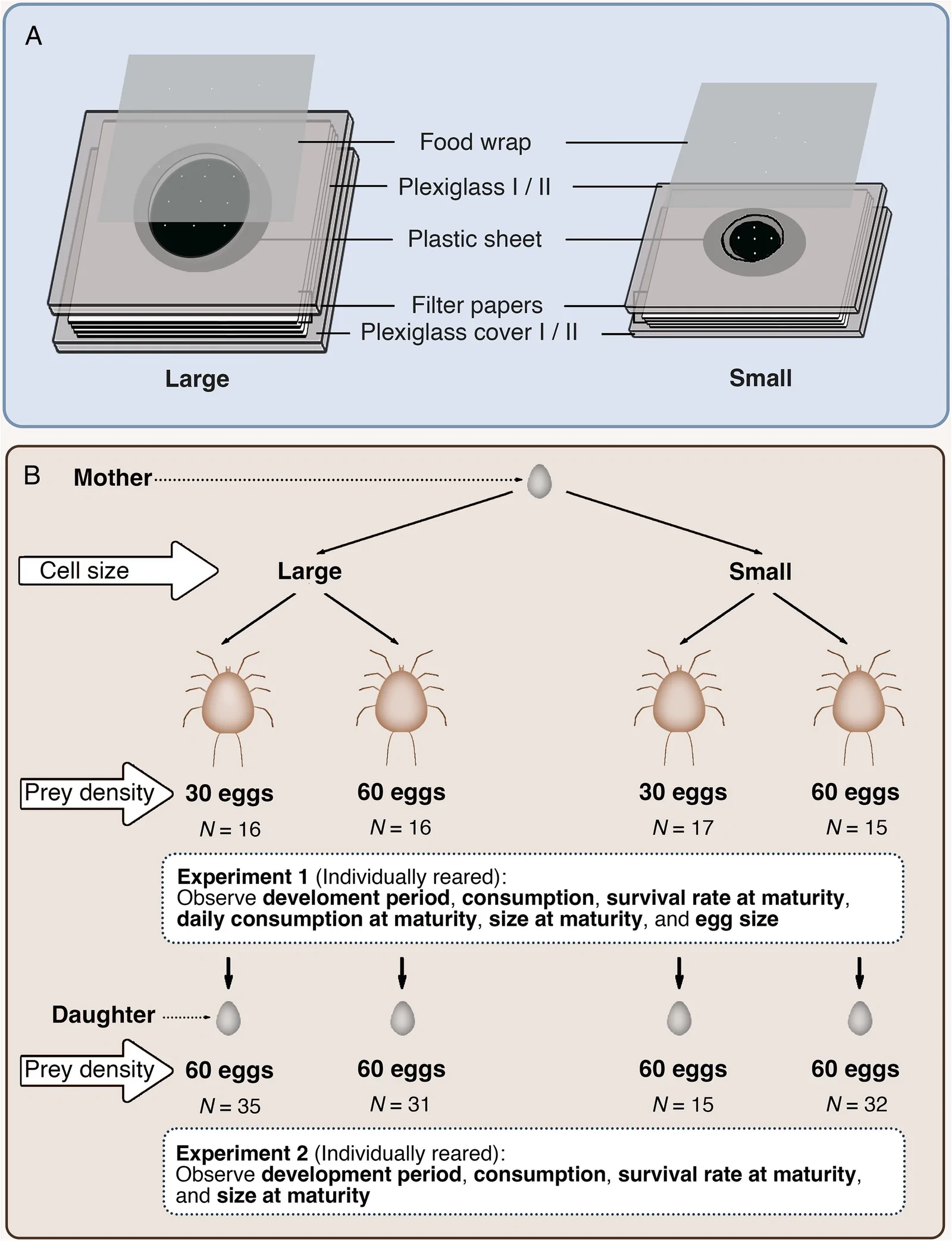
Rearing cell set-up and experimental procedures. Flowchart: “Section A—rearing cell structure; Section B—experimental design.”

In both cell types, the top of the chamber was sealed with food wrap perforated with 5 small holes (made using a size 0 insect pin) for ventilation. A black plastic disc (1.5 mm diameter) was placed at the base of the hole to enhance visibility of the mites. Beneath the disc, a stack of filter papers acted as a water reservoir. The disc was also perforated with 5 small holes to facilitate access to moisture. The bottom plexiglass slide (Plexiglass covers I and II) was positioned underneath the filter papers to retain moisture within the chamber. The 2 plexiglass components were secured using metal clips. The filter papers were saturated with filtered water daily during experiments to provide moisture.

### Experimental preparation

Approximately 30 *A. herbicolus* deutonymphs were collected from the laboratory colony to establish synchronized age cohorts. These individuals were provided with ad libitum access to live, mixed-stage *C. lactis* prey until they reached adulthood and commenced oviposition. Because *A. herbicolus* reproduces via thelytokous parthenogenesis, mating was not required for egg production (Zhang and Zhang 2022b).

To facilitate oviposition, sewing threads were introduced into the rearing containers to accommodate the species' known preference for laying eggs on hairy substrates (Zhang and Zhang 2022a; Liu et al. 2025). Approximately 5 days after cohort establishment, eggs (<16 h old) were collected and randomly assigned to treatments in Experiment 1.

### Experiment 1: Influence during maternal generation

Experiment 1 employed a 2 × 2 factorial design, incorporating 2 rearing cell sizes and 2 levels of prey availability (30 or 60 *C. lactis* eggs per cell) (Figure 1B). The freshly collected eggs were individually reared in either small or large cells. During immature development, each individual received either 30 or 60 previously frozen *C. lactis* eggs per cell (evenly distributed as 6 eggs per cluster) prior to hatching. Upon reaching adulthood, individuals were provided daily with prey corresponding to their initial prey density treatment—30 or 60 eggs per day. Following the onset of oviposition, females were allowed to lay eggs for 5 consecutive days. Prey consumption during development, survival rate to maturity, daily consumption of prey after maturity, with size at maturity and egg size measured using NIS-Elements software (version 5.10) (Figure 1B). The eggs produced during this period were subsequently collected and randomly allocated to further experiments (Experiment 2).

### Experiment 2: Influence on offspring

The newly collected eggs of *A. herbicolus* were measured under a phase-contrast microscope (Eclipse 90i, Nikon Corporation, Japan) using NIS-Elements (version 5.10). Egg sizes were estimated for individual volume (*V*) using the equation from Narushin (2005), using the length (*L*) and maximum breadth (*B*) of the eggs:


$$V\;=\;(0.6057\;-\;0.0018B)LB^{2}$$

These *A. herbicolus* eggs were subsequently reared individually in the small cells with 60 *C. lactis* eggs per cell as prey. Survival rate to adulthood, development period, prey consumption during development, and size at maturity (measured as dorsal plate length) were all examined (Figure 1B). Each treatment was replicated at least 15 times.

### Statistical analysis

All statistical analyses were performed using R (R Core Team 2024) in RStudio (version 2023.12.1). Data visualization was carried out with the *ggplot2* package (version 3.4.3) (Wickham 2016). Survival was analyzed using logistic regression with a binomial distribution. Generalized linear models (GLMs) with quasipoisson were used for development period, prey consumption during development, and daily prey consumption after maturity, due to the numeric results and right-skewed distribution. The GLMs with gaussian were used for size at maturity and egg size. The GLMs used rearing cell size and prey density as fixed factors and considering interaction between the fixed factors. Pairwise comparisons were conducted using the emmeans package (Lenth 2024). Data distribution and model overdispersion were examined prior to analysis. Overdispersion was detected in the count data; therefore, a quasipoisson error structure was applied where appropriate. For development period, size at maturity, prey consumption during development, daily consumption at maturity, and egg size, data were summarized as means with standard errors of the mean. Statistical significance was set at α = 0.05.

## Results

### Experiment 1: Influence during maternal generation

Survival rate was significantly influenced by prey availability (logistic regression: Wald χ^2^ = 9.375, df = 1, *P* = 0.002) but not by rearing cell size (Wald χ^2^ = 2.473, df = 1, *P* = 0.116). Individuals under high prey availability showed greater survival (Table 1). A significant interaction was found between prey availability and rearing cell size (Wald χ^2^ = 4.887, df = 1, *P* = 0.027): prey availability had a non-significant effect in large cells, whereas in small cells, survival was significantly lower under limited prey conditions.

**Table 1:** The survival rate, development period, size at maturity, and prey consumption during development for *Amblyseius herbicolus* under different treatments.

Cell	Prey density	*N*	Survival rate (%)	Developmental duration (days)	Size (µm)	Consumption
Small	30	17	35%^a^	6.1 ± 0.1^a^	355.0 ± 0.8^ab^	29.8 ± 0.2^a^
	60	15	87%^b^	6.2 ± 0.1^ab^	362.9 ± 3.3^a^	55.5 ± 1.5^c^
Large	30	16	63%^ab^	6.1 ± 0.1^a^	359.2 ± 2.1^b^	29.5 ± 0.5^a^
	60	16	63%^ab^	6.6 ± 0.2^b^	359.5 ± 3.2^ab^	43.0 ± 3.4^b^

^abc^Means followed by different letters denote significant differences from ART ANOVA pairwise comparisons (*P <* 0.05).

The developmental period of *A. herbicolus* was significantly influenced by prey availability (GLM: Wald χ^2^ = 12.367, df = 1, *P* < 0.001) but not by rearing cell size (Wald χ^2^ = 1.573, df = 1, *P* = 0.210). Individuals supplied with high prey availability (60 eggs per cell) had longer developmental durations (Table 1), but there was no significant interaction between prey availability and rearing cell size (Wald χ^2^ = 0.536, df = 1, *P* = 0.464).

Body size at maturity was significantly affected by prey availability (Wald χ^2^ = 3.935, df = 1, *P* = 0.047) but not by rearing cell size (Wald χ^2^ = 2.305, df = 1, *P* = 0.129). Individuals with higher prey availability reached larger adult size (Table 1). No interaction between prey availability and rearing cell size was detected (Wald χ^2^ = 0.094, df = 1, *P* = 0.759).

Prey consumption during development was significantly affected by both prey availability (GLM: Wald χ^2^ = 93.548, df = 1, *P* < 0.001) and rearing cell size (Wald χ^2^ = 14.829, df = 1, *P* < 0.001). Mites provided with high prey availability consumed more prey during development than those under low prey availability (Table 1). Within the high prey availability treatment, individuals reared in small cells consumed significantly more prey than those reared in large cells. A significant interaction between prey availability and rearing cell size was detected (Wald χ^2^ = 5.938, df = 1, *P* = 0.021): rearing cell size had little effect under low prey availability, but under high prey availability mites in small cells consumed substantially more prey than those in large cells.

Daily consumption at maturity was significantly affected by both prey availability (GLM: Wald χ^2^ = 6.141, df = 1, *P* = 0.013) and rearing cell size (Wald χ^2^ = 17.547, df = 1, *P* < 0.001). High prey availability and small rearing cell size treatments resulted in greater daily consumption (Table 2). An interaction was observed (Wald χ^2^ = 6.584, df = 1, *P* = 0.010): prey availability had no effect in large cells, whereas in small cells mites with abundant prey consumed more daily than those with limited prey.

**Table 2:** The adult daily prey consumption and egg size for *Amblyseius herbicolus* under different treatments.

Cell	Prey density	*n*	Daily consumption	*n*	Egg size (mm^3^)
Small	30	5	29.0 ± 0.7^a^	27	0.00138 ± 0.000002^b^
	60	9	36.4 ± 1.0^b^	38	0.00141 ± 0.000001^b^
Large	30	9	28.0 ± 0.8^a^	27	0.00138 ± 0.000001^b^
	60	10	28.1 ± 1.6^a^	33	0.00133 ± 0.000001^a^

^abc^Means followed by different letters denote significant differences from ART ANOVA pairwise comparisons (*P <* 0.05).

Egg size was significantly influenced by rearing cell size (GLM: Wald χ^2^ = 9.04, df = 1, *P* = 0.003) but not by prey availability (Wald χ^2^ = 1.738, df = 1, *P* = 0.187). Females in small cells produced larger eggs (Table 2). A significant interaction was found (Wald χ^2^ = 6.972, df = 1, *P* = 0.008): under high prey availability, egg size was greater in small cells, whereas rearing cell size had no effect under low prey conditions.

### Experiment 2: Influence on offspring

Survival rate to maturity was significantly influenced by maternal prey availability (logistic regression: Wald χ^2^ = 3.855, df = 1, *P* = 0.049) but not by maternal rearing cell size (Wald χ^2^ = 1.951, df = 1, *P* = 0.162). Offspring of mothers with high prey availability showed lower survival to maturity (Table 3). No interaction between maternal prey availability and rearing cell size was detected (Wald χ^2^ = 0.067, df = 1, *P* = 0.796).

**Table 3:** Survival rate, development period, size at maturity, and prey consumption during development for *Amblyseius herbicolus* under different maternal treatments.

Cell	Prey density	*N*	Survival rate (%)	Developmental duration (days)	Size (µm)	Consumption
Small	30	15	87%^b^	7.1 ± 0.2^a^	365.2 ± 3.5^b^	60.0 ± 0.0^b^
	60	32	59%^ab^	6.8 ± 0.2^a^	350.7 ± 2.4^a^	59.7 ± 0.2^b^
Large	30	35	69%^b^	6.6 ± 0.1^a^	356.2 ± 1.8^b^	50.6 ± 3.1^a^
	60	31	39%^a^	7.1 ± 0.1^a^	353.3 ± 3.1^ab^	59.2 ± 0.6^b^

All daughters were reared individually in small cells and provided with 60 *Carpoglyphus lactis* eggs per cell as prey.

^abc^Means followed by different letters denote significant differences from ART ANOVA pairwise comparisons (*P <* 0.05).

The developmental period of offspring was not significantly influenced by the prey availability of mothers (GLM: Wald χ^2^ = 0.049, df = 1, *P* = 0.824) or by maternal rearing cell size (Wald χ^2^ = 0.114, df = 1, *P* = 0.736). However, a significant interaction was detected between maternal prey availability and rearing cell size (Wald χ^2^ = 5.576, df = 1, *P* = 0.018). Maternal rearing cell size had no effect under low prey availability, whereas offspring of mothers kept in large cells with abundant prey tended to develop more rapidly (Table 3).

Body size at maturity was significantly affected by maternal prey availability (GLM: Wald χ^2^ = 10.908, df = 1, *P* < 0.001), but not by maternal rearing cell size (Wald χ^2^ = 0.001, df = 1, *P* = 0.978). Offspring of mothers exposed to high prey availability were smaller at maturity (Table 3). There was a significant interaction between maternal prey availability and rearing cell size (Wald χ^2^ = 3.949, df = 1, *P* = 0.047): prey availability had no effect in large cells, whereas in small cells, offspring of mothers under low prey availability were larger than those of mothers under high prey availability.

Prey consumption during development was significantly affected by maternal rearing cell size (GLM: Wald χ^2^ = 5.435, df = 1, *P* = 0.020), but not by maternal prey availability (Wald χ^2^ = 2.771, df = 1, *P* = 0.096). Offspring of mothers reared in large cells consumed fewer prey during development (Table 3). A marginally non-significant interaction was observed between maternal prey availability and rearing cell size (Wald χ^2^ = 3.834, df = 1, *P* = 0.050): under high prey availability maternal rearing cell size had little effect, but under low prey availability offspring of mothers from large cells consumed fewer prey.

## Discussion

This study examined how rearing cell size and prey availability influence the life-history traits of *A. herbicolus* within and across generations. The results supported our first hypothesis, but not the second one. Within the maternal generation, both rearing cell size and prey availability exerted strong influences on the performance of *A. herbicolus*. Individuals confined to small rearing cells consumed substantially more prey during both developmental and adult stages than those reared in large rearing cells. This elevated feeding possibly resulted from increased prey encounter rates within the restricted space, which could stimulate foraging activity and energy intake—a pattern consistent with previous studies on the phytoseiid predator *P. persimilis* (Kaiser 1983; Krips et al. 1999). Similar effects of spatial limitation on foraging efficiency have been reported in other predatory mites (Kaiser 1983) and arthropods (Uiterwaal and DeLong 2018), where smaller rearing cells enhanced prey detection and handling success. Nevertheless, greater prey consumption does not necessarily translate into shorter development, higher survival, or larger adult size; in our study only egg size increased, implying a disproportionately low conversion of ingested energy into growth or reproductive output when mites were reared in small rearing cells. Our results are consistent with previous studies of *Drosophila*, whereby restricted rearing condition elevate maintenance costs by increasing stress-related activity, thereby weakening the translation of additional prey consumption into growth or fecundity (Gordon and Masek 2021). Together, these results indicate that small rearing cells can increase the predation rate, but also impose physiological stress, elevating energy demands and driving excessive feeding without corresponding gains.

Survival differences between prey availability treatments were most pronounced in small rearing cells, where environmental stress was highest. The survival rates of mothers under low prey availability were much lower than those of the mothers under high prey availability. This finding suggests that while small rearing cell size elevates potential stress, sufficient prey supply may buffer its negative effects. This is consistent with a previous study, which found that sufficient food resources partially compensate for environmental stress in predator mites (Montserrat et al. 2013). Therefore, whereas restricted space may elevate maintenance costs, adequate prey sustains survival.

As expected, prey availability strongly influenced development, survival, and growth. Individuals provided with high prey availability developed faster, reached larger adult sizes, and consumed more prey than those reared under low prey availability. These patterns are consistent with resource-dependent plasticity widely observed in *A. herbicolus* and other phytoseiid mites (Zhang and Croft 1994; Walzer and Schausberger 2013; Han et al. 2022; Zhang and Zhang 2025), reinforcing that energy acquisition during development determines growth rate and survival.

The results did not support the second hypothesis: mothers living in smaller rearing cells with limited prey availability had better offspring compared with other treatments. Only the daughters of mothers maintained in large rearing cells with limited prey availability exhibited lower prey consumption during development, yet their size at maturity did not differ significantly from others, suggesting higher energy conversion efficiency or reduced developmental feeding; in contrast, daughters of mothers kept in small rearing cells showed higher developmental consumption, indicating that maternal conditions can influence offspring feeding behavior across generations (Zhang et al. 2025). Moreover, offspring of mothers exposed to limited prey exhibited higher survival rates and larger body sizes than those of mothers provided with abundant prey. This pattern probably results from maternal effects in which offspring phenotypes are shaped by maternal nutritional experience, potentially conferring adaptive advantages under tough environmental constraints (Marshall and Uller 2007). Another possible explanation is that mild confinement in small cells may have induced a form of transgenerational hormesis, whereby exposure to moderate environmental challenges enhances tolerance and physiological performance in subsequent generations (Calabrese and Mattson 2017).

An interaction between prey availability and rearing cell size was also evident in reproductive allocation, as reflected by egg size. Under low prey availability, egg size did not differ significantly between mothers in small and large cells. However, under high prey availability, mothers in small cells tended to produce larger eggs than those in large cells. This indicates that confinement can influence reproductive investment, even when resources are abundant. Mothers in larger, resource-rich rearing cells may perceive the environment as favorable and reduce per-egg investment, whereas those in smaller, constrained environments allocate relatively more resources to each egg to enhance offspring survival under poor conditions. Such plastic trade-offs between egg size and fecundity are considered adaptive responses to environmental variability (Marshall and Uller 2007), consistent with findings in *Phytoseiulus persimilis* (Walzer and Schausberger 2015) and other arthropods (Fox and Czesak 2000).

This study has several limitations that should be considered in further work. First, the rearing cell size and prey availability used for the offspring generation did not correspond exactly to those experienced by their mothers. This mismatch may have influenced the offspring's adaptive responses and the extent of observed maternal effects, as environmental continuity between generations is often critical for the expression of adaptive transgenerational plasticity (Marshall and Uller 2007). Second, prey density varied among treatments due to differences in both rearing cell size and prey availability. Specifically, in small rearing cells, prey density was 0.027/mm^2^ with low prey availability, and 0.054/mm^2^ with high prey availability. In large rearing cells, prey density was 0.01/mm^2^ with low prey availability, and 0.02/mm^2^ with high prey availability. Such variation may have introduced additional complexity to the interpretation of treatment effects, as differences in prey encounter rates and feeding opportunities could influence energy acquisition and behavioral responses across treatments.

Overall, this study demonstrates that both rearing environment and prey abundance can shape individual and offspring performance in *A. herbicolus*. While small rearing environments elevated prey consumption and egg investment during the maternal generation, prey limitation experienced by mothers appears to induce compensatory advantages in offspring. These results underscore the importance of considering both within- and across-generation influences when interpreting the life-history responses of predatory mites. Future research should investigate the physiological mechanisms underlying these maternal effects, particularly the potential roles of nutrient allocation and epigenetic regulation. Enclosure size for both individual rearing and mass rearing of invertebrate species should be considered.

## Data Availability

All data and analyses are available upon request to the authors.

## Artificial intelligence (AI)-assisted technologies

Artificial intelligence–assisted tools were used solely to improve the grammar of the manuscript. These tools did not contribute to the study design, data collection, data analysis, interpretation of results, or generation of scientific conclusions.

## References

[zoag008-B1] Amano H, Chant DA, 1977. Life history and reproduction of two species of predacious mites, *Phytoseiulus persimilis* Athias-Henriot and *Amblyseius andersoni* (Chant) (Acarina: Phytoseiidae). Can J Zool 55(12):1978–1983.

[zoag008-B2] Amano H, Chant DA, 1978. Some factors affecting reproduction and sex ratios in two species of predacious mites, *Phytoseiulus persimilis* Athias-Henriot and *Amblyseius andersoni* (Chant) (Acarina: Phytoseiidae). Can J Zool 56(7):1593–1607.

[zoag008-B3] An W, Zhang Y, Zhou A, Hu Y, 2021. Detrimental effects of restricted cage size on reproductive performance, exploration ability, and anxiety but not working memory of Kunming mice. Front Behav Neurosci 15:651782. 10.3389/fnbeh.2021.651782 PMC8248795 34220461

[zoag008-B4] Anastasiadi D, Venney CJ, Bernatchez L, Wellenreuther M, 2021. Epigenetic inheritance and reproductive mode in plants and animals. Trends Ecol Evol 36(12):1124–1140. 10.1016/j.tree.2021.08.006 34489118

[zoag008-B5] Bell AM, Hellmann JK, 2019. An integrative framework for understanding the mechanisms and multigenerational consequences of transgenerational plasticity. Annu Rev Ecol Evol Syst 50(1):97–118. 10.1146/annurev-ecolsys-110218-024613 PMC9427003 36046014

[zoag008-B6] Bonduriansky R , 2012. Rethinking heredity, again. Trends Ecol Evol 27(6):330–336. 10.1016/j.tree.2012.02.003 22445060

[zoag008-B7] Bonduriansky R, Crean AJ, Davey M, 2018. What are parental condition-transfer effects and how can they be detected? Methods Ecol Evol 9(3):450–456.

[zoag008-B8] Bonduriansky R, Day T, 2009. Nongenetic inheritance and its evolutionary implications. Annu Rev Ecol Evol Syst 40(1):103–125.

[zoag008-B9] Bosua HJ, Nicolson SW, Archer CR, Pirk CWW, 2018. Effects of cage volume and bee density on survival and nutrient intake of honeybees (*Apis mellifera* L.) under laboratory conditions. Apidologie 49(6):734–746.

[zoag008-B10] Bussolini LT, Crates R, Magrath MJL, Stojanovic D, 2023. Identifying factors affecting captive breeding success in a critically endangered species. Emu Austral Ornithol 123(2):161–169.

[zoag008-B11] Calabrese EJ, Mattson MP, 2017. How does hormesis impact biology, toxicology, and medicine? NPJ Aging Mech Dis 3(1):13. 10.1038/s41514-017-0013-z PMC5601424 28944077

[zoag008-B12] Carere C, Mather J, editors. 2019. The Welfare of Invertebrate Animals. Cham: Springer.

[zoag008-B13] Cavalcante ACC, dos Santos VLV, Rossi LC, de Moraes GJ, 2015. Potential of five Brazilian populations of Phytoseiidae (Acari) for the biological control of *Bemisia tabaci* (Insecta: Hemiptera). J Econ Entomol 108(1):29–33. 10.1093/jee/tou003 26470100

[zoag008-B14] Crates R, Stojanovic D, Heinsohn R, 2023. The phenotypic costs of captivity. Biol Rev Camb Philos Soc 98(2):434–449. 10.1111/brv.12913 36341701

[zoag008-B15] Fox CW, Czesak ME, 2000. Evolutionary ecology of progeny size in arthropods. Annu Rev Entomol 45(1):341–369. 10.1146/annurev.ento.45.1.341 10761581

[zoag008-B16] Gordon J, Masek P, 2021. Excessive energy expenditure due to acute physical restraint disrupts *Drosophila* motivational feeding response. Sci Rep 11(1):24208. 10.1038/s41598-021-03575-3 PMC8683507 34921197

[zoag008-B17] Groothuis TGG, Kumar N, Hsu B-Y, 2020. Explaining discrepancies in the study of maternal effects: the role of context and embryo. Curr Opin Behav Sci 36:185–192.

[zoag008-B18] Han X, Zhang K, Chen X, Zhang Z-Q, 2024. Diet restriction in parents shapes the lives of their offspring: a laboratory study using a predatory mite (Acari: Phytoseiidae). Syst Appl Acarol 29:45–59.

[zoag008-B19] Han X, Zhang K, Xu Y, Zhang Z-Q, 2022. Prey requirement and development of a predatory mite under diet restriction: *Phytoseiulus persimilis* Athias-Henriot (Phytoseiidae) feeding on *Tetranychus urticae* Koch (Tetranychidae). Syst Appl Acarol 27:2103–2110.

[zoag008-B20] Kaiser H , 1983. Small-scale spatial heterogeneity influences predation success in an unexpected way: model experiments on the functional response of predatory mites (Acarina). Oecologia 56(2-3):249–256. 10.1007/BF00379698 28310202

[zoag008-B21] Kalile MO, Cardoso AC, Pallini A, Fonseca MM, Elliot SL et al, 2021. A predatory mite as potential biological control agent of *Diaphorina citri*. BioControl 66(2):237–248.

[zoag008-B22] Kavousi A, Chi H, Talebi K, Bandani A, Ashouri A et al, 2009. Demographic traits of *Tetranychus urticae* (Acari: Tetranychidae) on leaf discs and whole leaves. J Econ Entomol 102(2):595–601. 10.1603/029.102.0217 19449639

[zoag008-B23] Krips OE, Kleijn PW, Willems PEL, Gols GJZ, Dicke M, 1999. Leaf hairs influence searching efficiency and predation rate of the predatory mite *Phytoseiulus persimilis* (Acari: Phytoseiidae). Exp Appl Acarol 23(2):119–131.

[zoag008-B24] Kuijper B, Johnstone RA, 2018. Maternal effects and parent–offspring conflict. Evolution 72(2):220–233. 10.1111/evo.13403 29210448

[zoag008-B25] Lam W, Paynter Q, Zhang Z-Q, 2021. Functional response of *Amblyseius herbicolus* (Acari: Phytoseiidae) on *Sericothrips staphylinus* (Thysanoptera: Thripidae), an ineffective biocontrol agent of gorse. Biol Control 152:104468.

[zoag008-B26] Lenth RV , 2024. emmeans: Estimated marginal means, aka least-squares means. R package version 1.10.0. Vienna: R Foundation for Statistical Computing. https://CRAN.R-project.org/package=emmeans.

[zoag008-B27] Lewbart GA, Zachariah TT, 2023. Aquatic and terrestrial invertebrate welfare. Animals (Basel) 13(21):3375. 10.3390/ani13213375 PMC10649180 37958134

[zoag008-B28] Liu Z, Zhang K, Zhang Z, 2024. Enhancing the efficiency of egg collection of the astigmatid mite *Carpoglyphus lactis* (Acari: Carpoglyphidae) as a diet for predatory mites. Syst Appl Acarol 29:355–358.

[zoag008-B29] Liu Z, Zhang K, Zhang Z-Q, 2025. Phototactic behaviour and oviposition of seven species of Phytoseiidae (Acari: Mesostigmata). Pest Manag Sci 81(4):1765–1770. 10.1002/ps.8575 39632773

[zoag008-B30] Ma M, Zhang K, Fan Q-H, Zhang Z-Q, 2024. Description of ontogenetic changes in the morphology of *Amblyseius herbicolus* (Chant, 1959) and *Amblyseius lentiginosus* Denmark & Schicha, 1974 (Acari: Phytoseiidae). Zootaxa 5485(1):7–37. 10.11646/zootaxa.5485.1.4 39646004

[zoag008-B31] Marshall D, Uller T, 2007. When is a maternal effect adaptive? Oikos 116(12):1957–1963.

[zoag008-B32] Mason GJ , 2010. Species differences in responses to captivity: stress, welfare and the comparative method. Trends Ecol Evol 25(12):713–721. 10.1016/j.tree.2010.08.011 20952089

[zoag008-B33] McMurtry JA, de Moraes GJ, Sourassou NF, 2013. Revision of the lifestyles of phytoseiid mites (Acari: Phytoseiidae) and implications for biological control strategies. Syst Appl Acarol 18:297–320.

[zoag008-B34] McMurtry JA, Sourassou NF, Demite PR, 2015. The Phytoseiidae (Acari: Mesostigmata) as biological control agents. In: Carrillo D, de Moraes GJ, Peña JE, editors. Prospects for Biological Control of Plant Feeding Mites and Other Harmful Organisms. Cham: Springer, 133–149.

[zoag008-B35] Montserrat M, Guzmán C, Sahún RM, Belda JE, Hormaza JI, 2013. Pollen supply promotes, but high temperatures demote, predatory mite abundance in avocado orchards. Agric Ecosyst Environ 164:155–161.

[zoag008-B36] Morgan KN, Tromborg CT, 2007. Sources of stress in captivity. Appl Anim Behav Sci 102(3-4):262–302.

[zoag008-B37] Narushin VG , 2005. Egg geometry calculation using the measurements of length and breadth. Poult Sci 84(3):482–484. 10.1093/ps/84.3.482 15782919

[zoag008-B38] Perdue BM, Sherwen SL, Maple TL, 2020. Editorial: the science and practice of captive animal welfare. Front Psychol 11:1851. 10.3389/fpsyg.2020.01851 PMC7457122 32922329

[zoag008-B39] Pollo S, Vitale A, 2019. Invertebrates and humans: science, ethics, and policy. In: Carere C, Mather J, editors. The Welfare of Invertebrate Animals. Cham: Springer, 7–22.

[zoag008-B40] Räsänen K, Kruuk LEB, 2007. Maternal effects and evolution at ecological timescales. Funct Ecol 21(3):408–421.

[zoag008-B41] R Core Team , 2024. R: A Language and Environment for Statistical Computing. Vienna: R Foundation for Statistical Computing. http://www.R-project.org/

[zoag008-B42] Reis PR, Teodoro AV, Pedro Neto M, da Silva EA, 2007. Life history of *Amblyseius herbicolus* (Chant) (Acari: Phytoseiidae) on coffee plants. Neotrop Entomol 36(2):282–287. 10.1590/s1519-566x2007000200016 17607463

[zoag008-B43] Uiterwaal SF, DeLong JP, 2018. Multiple factors, including arena size, shape the functional responses of ladybird beetles. J Appl Ecol 55(5):2429–2438.

[zoag008-B44] Walzer A, Schausberger P, 2013. Intra- and trans-generational costs of reduced female body size caused by food limitation early in life in mites. PLoS One 8(11):e79089. 10.1371/journal.pone.0079089 PMC3827130 24265745

[zoag008-B45] Walzer A, Schausberger P, 2015. Food stress causes sex-specific maternal effects in mites. J Exp Biol 218(Pt\ 16):2603–2609. 10.1242/jeb.123752 PMC4550958 26089530

[zoag008-B46] Wang J, Zhang K, Li L, Zhang Z, 2024. Development and reproduction of four predatory mites (Parasitiformes: Phytoseiidae) feeding on the spider mites *Tetranychus evansi* and *T. urticae* (Trombidiformes: Tetranychidae) and the dried fruit mite *Carpoglyphus lactis* (Sarcoptiformes: Carpoglyphidae). Syst Appl Acarol 29:269–284.

[zoag008-B47] Wickham H , 2016. ggplot2: Elegant Graphics for Data Analysis. 2^nd^ ed. New York: Springer-Verlag. https://ggplot2-book.org/

[zoag008-B48] Widowski TM, Caston LJ, Hunniford ME, Cooley L, Torrey S, 2017. Effect of space allowance and cage size on laying hens housed in furnished cages. Part I: performance and well-being. Poult Sci 96(11):3805–3815. 10.3382/ps/pex197 PMC5850468 29050408

[zoag008-B49] Xie L, Yan Y, Zhang Z-Q, 2018. Development, survival and reproduction of *Stratiolaelaps scimitus* (Acari: Laelapidae) on four diets. Syst Appl Acarol 23:779–794.

[zoag008-B50] Xin T-R, Zhang Z-Q, 2021. Suitability of pollen as an alternative food source for different developmental stages of *Amblyseius herbicolus* (Chant) (Acari: Phytoseiidae) to facilitate predation on whitefly eggs. Acarologia 61(4):790–801.

[zoag008-B51] Xu Y, Zhang K, Han X, Zhang Z-Q, 2025. Early life food intake modulates effects of diet restriction on lifespan and fecundity in later life in a predatory mite (Acari: Phytoseiidae). Curr Zool 71(3):295–303. 10.1093/cz/zoae047 PMC12227415 40620589

[zoag008-B52] Xu Y, Zhang K, Zhang Z-Q, 2023. The impact of conspecific presence on diet-induced developmental responses in a predatory mite. Syst Appl Acarol 28:1705–1715.

[zoag008-B53] Zhang Z , 2003a. Mites of Greenhouses: Identification, Biology and Control. Wallingford: CABI.

[zoag008-B54] Zhang Z , 2003b. Phytoseiid mites. In: Zhang Z, editor. Mites of Greenhouses: Identification, Biology and Control. Wallingford: CABI, 171–179.

[zoag008-B55] Zhang Z-Q, Croft BA, 1994. A comparative life-history study of immature *Amblyseius fallacis*, *A. andersoni*, *Typhlodromus occidentalis* and *T. pyri* (Acari: Phytoseiidae) with a review of larval feeding patterns in the family. Exp Appl Acarol 18(11-12):631–657.

[zoag008-B56] Zhang K, Schausberger P, Holwell G, Zhang Z, 2025. An inverse Lansing effect: older mothers produce offspring with improved development and growth efficiency. bioRxiv 2025-10. 10.1101/2025.10.16.682747

[zoag008-B57] Zhang K, Zhang Z-Q, 2021. The dried fruit mite *Carpoglyphus lactis* (Acari: Carpoglyphidae) is a suitable alternative prey for *Amblyseius herbicolus* (Acari: Phytoseiidae). Syst Appl Acarol 26:2167–2176.

[zoag008-B58] Zhang K, Zhang Z-Q, 2022a. *Amblyseius herbicolus* mothers prefer to oviposit near eggs of non-kin in the absence of prey *Carpoglyphus lactis* (Acari: Phytoseiidae, Carpoglyphidae). Syst Appl Acarol 27:2347–2354.

[zoag008-B59] Zhang K, Zhang Z-Q, 2022b. Kin recognition by cannibals is modulated by hunger level in a generalist predatory mite *Amblyseius herbicolus* (Chant) (Acari: Phytoseiidae). J Appl Entomol 146(5):579–585.

[zoag008-B60] Zhang K, Zhang Z, 2025. Diet-induced developmental and morphological plasticity in a thelytokous predatory mite *Amblyseius herbicolus* (Chant) (Acari: Phytoseiidae). Ecol Evol 15(10):e72280. 10.1002/ece3.72280 PMC12516015 41089887

